# Traducción y adaptación transcultural al contexto español del cuestionario *Iceland-Family Perceived Support Questionnaire*

**DOI:** 10.23938/ASSN.1144

**Published:** 2025-11-19

**Authors:** Bilal Benbelkheir Núñez, Cristina Alfaro-Díaz, María Pueyo-Garrigues, María Carrión Torre, Nuria Esandi Larramendi

**Affiliations:** 1 Departamento de Enfermería de la Persona Adulta Facultad de Enfermería Universidad de Navarra Pamplona España; 2 Instituto de Investigación Sanitaria de Navarra (IdiSNA) Pamplona España; 3 Departamento de Enfermería Comunitaria y Materno-Infantil Facultad de Enfermería Universidad de Navarra Pamplona España; 4 Clínica Universidad de Navarra Madrid España

**Keywords:** Traducción, Adaptación transcultural, Apoyo familiar, Enfermería, Cuestionarios, Translation, Cross-Cultural Adaptation, Family Support, Nursing, Questionnaires

## Abstract

**Fundamento:**

Las enfermeras desempeñan un rol fundamental en el apoyo a las familias de pacientes hospitalizados, especialmente con enfermedades crónicas. Mejorar la calidad del cuidado requiere instrumentos validados que evalúen la percepción del apoyo familiar. El *Iceland-Family Perceived Support Questionnaire* (ICE-FPSQ) mide esta percepción desde la perspectiva familiar. Sin embargo, no existe una versión adaptada culturalmente al español. Este estudio tiene como objetivos i) desarrollar una versión del ICE-FPSQ específica para enfermeras; y ii) traducir y adaptar conceptual y lingüísticamente ambos instrumentos al contexto español.

**Metodología:**

Se siguieron cuatro fases: I) adaptación del ICE-FPSQ para enfermeras; II) traducción, retrotraducción y adaptación lingüística de ambos instrumentos; III) validación mediante panel de expertos; y IV) estudio piloto con técnicas de entrevista cognitiva para evaluar aplicabilidad y comprensión.

**Resultados:**

Se obtuvieron dos versiones traducidas y adaptadas del ICE-FPSQ, una para familiares y otra para enfermeras. En términos de relevancia, la validez de contenido de los instrumentos fue excelente (S-CVI/AVE =1). En cuanto a la claridad, el índice de validez de contenido fue 0,77 en la versión para familiares, y 0,88 en la versión para enfermeras. Los participantes del estudio piloto destacaron la claridad y la rapidez en su cumplimentación.

**Conclusiones:**

Se han obtenido dos herramientas culturalmente adaptadas al contexto español, con adecuada claridad y relevancia para evaluar la percepción del apoyo tanto desde la perspectiva familiar como de las enfermeras. Los resultados respaldan su idoneidad para futuras validaciones psicométricas en el contexto español.

## Abstract (en)

**Background:**

Nurses play a crucial role in providing family support to families of hospitalized patients, particularly in the context of chronic diseases. To enhance the quality of care validated tools are needed to assess perceived family support. The Iceland-Family Perceived Support Questionnaire (ICE-FPSQ) measures families’ perceptions of support provided by nurses; however, no culturally adapted Spanish version exists. The objectives of the study are: i) to develop a version of the ICE-FPSQ suitable for nurses; and ii) to translate and adapt both instruments conceptually and linguistically to the Spanish context.

**Methodology:**

The study followed four phases: I) adaptation of the ICE-FPSQ for nurses; II) translation, back-translation, and linguistic adaptation of both scales; III) content validation by an expert panel; and IV) a pilot study with cognitive interview techniques to assess the applicability and comprehension.

**Results:**

Translated and adapted versions were obtained: one for family members and another for nurses. In terms of relevance, content validity was excellent for both instruments (S-CVI/AVE = 1). In terms of clarity, the family version achieved a content validity index of 0.77, while the nurses’ version reached 0.88. Pilot study participants highlighted the clarity of the items and the speed of completion.

**Conclusions:**

Two culturally adapted tools for the Spanish context have been developed, demonstrating adequate clarity and relevance for assessing perceived support from both family and nurse perspectives. These findings indicate that both instruments are suitable for their future psychometric validation in Spain.

## INTRODUCCIÓN

La literatura científica aporta evidencia consistente de que el impacto de la enfermedad y su tratamiento trasciende al paciente, afectando significativamente a su red de apoyo, concretamente a sus familiares más cercanos^1^. Las familias no solo asumen responsabilidades en torno al cuidado de su ser querido, sino que experimentan elevados niveles de ansiedad, incertidumbre y estrés emocional, derivados tanto de la preocupación por la evolución clínica de su familiar como de las exigencias inherentes al proceso de cuidados^2^.

Este impacto se intensifica si la enfermedad requiere hospitalización, un evento altamente estresante que genera desafíos emocionales, sociales y económicos para toda la unidad familiar^3^. En este contexto, el apoyo familiar brindado por las enfermeras (entendidas como personal de enfermería de ambos sexos) se convierte en un componente esencial de la atención, ya que contribuye a mitigar el impacto de la hospitalización y, con ello, a disminuir el sufrimiento familiar y promover el bienestar de todos los involucrados^4^. De hecho, estudios recientes han identificado que la percepción de las familias sobre el apoyo por parte de profesionales de la salud influye positivamente en su capacidad de afrontamiento y en su proceso de toma de decisiones, reduciendo el estrés y favoreciendo una mejor adaptación a la enfermedad y la hospitalización del ser querido^5^^,^^6^. Garantizar un adecuado apoyo familiar es una estrategia fundamental para optimizar los resultados en salud y minimizar las consecuencias psicosociales a largo plazo^7^^,^^8^.

A menudo, las enfermeras consideran que sus intervenciones responden de manera eficaz a las necesidades de apoyo de las familias^9^. Esta percepción puede enriquecerse si se incorpora la perspectiva de las propias familias^10^. Comprender cómo las familias perciben el apoyo y los cuidados proporcionados por las enfermeras permite evaluar si las intervenciones realmente satisfacen las necesidades de apoyo de pacientes y familias. Para ello, se han desarrollado y validado diversos instrumentos que miden la percepción familiar del apoyo y cuidado proporcionado por las enfermeras, entre los que destacan tres de ellos: el *Nursing Parent Support tool*^11^ mide el apoyo emocional, informativo y tangible que los padres reciben de las enfermeras en contextos pediátricos; el *Family-Centered Care Scale in Intensive Care Unit*^12^ evalúa en unidades de cuidados intensivos la percepción de la familia sobre el apoyo recibido (comunicación, inclusión en toma de decisiones y respeto) en unidades de cuidados intensivos; y el *Iceland-Family Perceived Support Questionnaire* (ICE-FPSQ)^13^ evalúa la percepción de la familia en el contexto de cuidado de un familiar enfermo.

El ICE-FPSQ se ha utilizado en distintos contextos como plantas de hospitalización^5^, unidades de cuidados intensivos^14^ o unidades de hospital de día^15^, aplicado tanto en población tanto adulta^16^ como pediátrica^13^, y en el marco de distintas enfermedades, entre ellas el cáncer^5^ y las cardiopatías congénitas^17^. El ICE-FPSQ se ha consolidado como una herramienta valiosa para evaluar la percepción familiar del apoyo proporcionado por el personal de enfermería.

A pesar de que el ICE-FPSQ se ha traducido a distintos idiomas (sueco^17^, danés^18^, portugués^15^ y alemán^16^), actualmente no existe una versión en español. Además, aunque su utilidad está ampliamente reconocida, el ICE-FPSQ se centra exclusivamente en la percepción de las familias, sin considerar la perspectiva de las propias enfermeras. Disponer de instrumentos que evalúen la percepción tanto de familiares como del personal enfermero permitiría analizar de manera más completa cómo se brinda el apoyo y el cuidado a la familia en la práctica clínica, permitiendo identificar incluso posibles divergencias entre ambas perspectivas^19^.

Por tanto, resulta necesario desarrollar una nueva versión que evalúe la percepción de las enfermeras sobre el apoyo que proporcionan a la familia. Por ello, los objetivos del presente estudio son obtener una versión del ICE-FPSQ adaptada para su uso en enfermeras, y traducir y adaptar conceptual y lingüísticamente al contexto español el ICE-FPSQ y su versión para enfermeras.

## MATERIAL Y MÉTODOS

Se llevó a cabo un proceso sistemático de adaptación transcultural de los instrumentos en varias fases: adaptación conceptual del cuestionario original creando una nueva versión para enfermeras, traducción y retrotraducción de ambos instrumentos, validación de contenido mediante panel de expertos y estudio piloto con entrevistas cognitivas.

### Descripción del ICE-FPSQ

La versión original del instrumento *Iceland-Family Perceived Support Questionnaire* (ICE-FPSQ) fue desarrollada en inglés en 2007 por Svavarsdottir. Este instrumento fue diseñado para evaluar la percepción de la familia respecto al apoyo recibido por parte de las enfermeras en el contexto de cuidado de un familiar enfermo, y ha sido ampliamente validado y utilizado en distintos contextos culturales. Su base teórica se fundamenta en el Modelo Calgary de Intervención Familiar (*Calgary Family Intervention Model*, CFIM), que conceptualiza a paciente y familia como una unidad de cuidado y enfatiza la necesidad de ofrecer intervenciones de apoyo dirigidas a cada una de las dimensiones del funcionamiento familiar: cognitiva (proporcionar información y apoyo en la toma de decisiones), afectiva (validar y normalizar respuestas emocionales) y actitudinal (fortalecer la resiliencia y el afrontamiento familiar)^13^^,^^20^, promoviendo su empoderamiento a partir de sus fortalezas familiares^21^. Ha demostrado ser un instrumento válido y fiable para medir la percepción de la familia sobre el apoyo recibido por parte de las enfermeras, con coeficientes alfa de Cronbach superiores a 0,9 en distintas validaciones^5^^,^^15^^,^^17^.

Tras la validación del instrumento, se confirmó que está compuesto por dos subescalas:

- apoyo cognitivo: mide el grado en que los familiares perciben que reciben información clara, compresible y útil; incluye los ítems de la dimensión cognitiva del funcionamiento familiar;
- apoyo emocional: mide la percepción sobre el apoyo que las enfermeras ofrecen a las familias para ayudar a manejar la carga emocional asociada con la enfermedad de su ser incluye las dimensiones afectiva y actitudinal del funcionamiento familiar porque los ítems relacionados con estas dimensiones mostraron una alta correlación en el análisis factorial.

El ICE-FPSQ consta de 14 ítems que se responden con una escala tipo Likert de cinco puntos (1 casi nunca hasta 5 casi siempre). La puntuación total de la escala varía entre 14 y 70 puntos, donde una puntuación mayor indica mayor percepción del apoyo recibido por las enfermeras. Este cuestionario presenta buenas propiedades psicométricas, con valores de alfa de Cronbach superiores a 0,9 en distintas validaciones^5^^,^^15^^,^^17^, de 0,953 para la escala total, 0,874 para la subescala de apoyo cognitivo y 0,937 para la subescala de apoyo emocional.

*Fase 1. Adaptación del instrumento para su uso en enfermeras*

El ICE-FPSQ es un instrumento diseñado para medir el apoyo percibido por la familia. En el presente estudio se ha llevado a cabo una adaptación de este instrumento para evaluar la percepción de las enfermeras respecto al apoyo ofrecido a las familias. Esta adaptación se llevó a cabo entre los autores del presente estudio y las autoras del instrumento original (ICE-FPSQ).

En primer lugar, se realizó una reunión con las autoras del instrumento, para presentarles el objetivo del estudio y obtener su autorización para llevar a cabo la traducción y adaptación del ICE-FPSQ al contexto español. Tras ello, se profundizó en el CFIM, que constituye la base conceptual del instrumento original, con el propósito de asegurar que la versión adaptada mantuviera el constructo central de *apoyo familiar*. Este proceso incluyó la revisión de los elementos teóricos y prácticos del modelo, garantizando la preservación de las dos subescalas del instrumento -apoyo cognitivo y apoyo funcional-, así como de sus principios fundamentales. De este modo, se prestó especial atención a que la adaptación respetara tanto la estructura del instrumento como la coherencia conceptual, de forma que los ítems continuaran reflejando con precisión la experiencia del apoyo familiar^1^.

A continuación, el equipo investigador trabajó en el desarrollo de una nueva versión del cuestionario, específicamente adaptada para su aplicación en enfermeras, *The Health Care Professionals Perception regarding Offering Family Support Questionnaire* (HCPP-OFSQ). El equipo investigador redefinió los ítems para incluir la perspectiva enfermera, manteniendo la misma estructura, número y contenido de los ítems originales, para preservar la equivalencia funcional y métrica con el ICE-FPSQ.

La versión adaptada se envió a las autoras del instrumento original, quienes proporcionaron retroalimentación por escrito con observaciones y sugerencias para mejorar la redacción de los distintos ítems. Posteriormente, se realizó una reunión en línea entre el equipo investigador y las autoras del instrumento para analizar comparativamente los ítems de la versión original y de la versión adaptada para enfermeras. Durante esta sesión, se discutió la pertinencia del nombre del instrumento, se revisaron y ajustaron algunos ítems para garantizar su equivalencia conceptual desde la perspectiva profesional, y se clarificaron las instrucciones para su adecuada cumplimentación. No se añadieron ítems ni se modificó la estructura dimensional del cuestionario en aras de mantener la equivalencia funcional y métrica de las versiones que permita futuras comparaciones entre ambas poblaciones.

Este proceso permitió garantizar una adaptación rigurosa y alineada con la versión original, asegurando que el instrumento mantuviera su validez y relevancia en el nuevo contexto de aplicación.

*Fase 2. Traducción y adaptación transcultural de los instrumentos*

**Figura 1:**
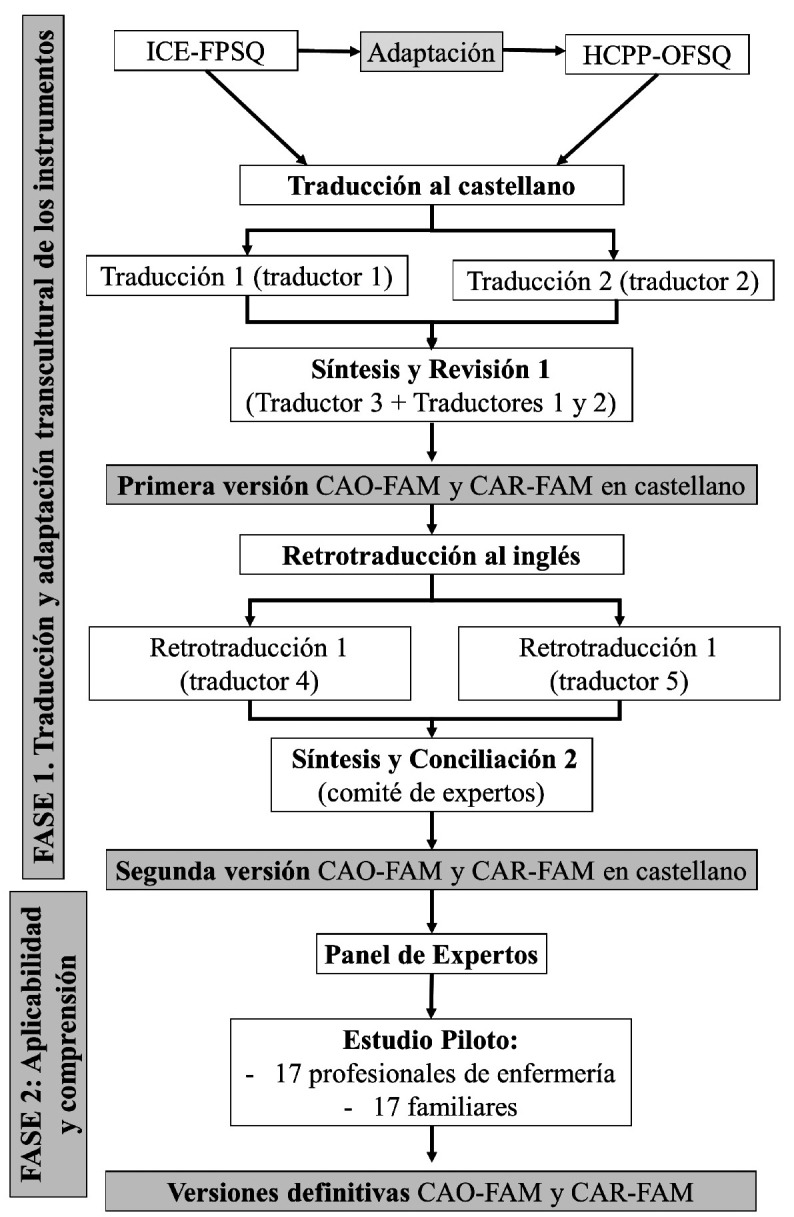
Diagrama de flujo del proceso de traducción y adaptación del *Iceland-Family Perceived Support Questionnaire* y del *Health Care Profesionals Perception regarding Offering Family Support Questionnaire* al castellano.

ICE-FPSQ: *Iceland-Family Perceived Support Questionnaire*; HCPP-OFSQ: *Health Care Professionals Perception regarding Offering Family Support Questionnaire*; CAO-FAM: Cuestionario sobre la percepción de los profesionales de la salud del apoyo ofrecido a la familia; CAR-FAM: Cuestionario sobre la percepción familiar del apoyo recibido por los profesionales de la salud.

Para llevar a cabo el proceso de traducción y adaptación transcultural se siguió la guía *Translation, adaptation and validation of instruments or scales for use in cross-cultural health care research: a clear and user-friendly guideline* elaborada por Sousa y Rojjanasrirat^22^, que coincide con las directrices principales formuladas por la Organización Mundial de la Salud^23^ para la traducción y retrotraducción de instrumentos (Fig. 1).

En primer lugar, se llevaron a cabo dos traducciones independientes de cada una de las versiones originales del ICE-FPSQ y del HCPP-OFSQ (Tabla 1) por dos personas bilingües, cuya lengua materna era el castellano. La primera traductora era una enfermera experta en el contenido del instrumento, con amplio conocimiento en el área del cuidado a la familia; la segunda traductora, también enfermera, estaba familiarizada con expresiones idiomáticas, frases coloquiales y modismos del inglés. Como resultado de este primer paso se obtuvieron dos versiones traducidas al castellano de cada uno de los dos instrumentos.

Posteriormente, una tercera traductora comparó las versiones y estas, a su vez, con la escala original para identificar discrepancias en palabras, frases o significados. Esta tercera traductora era una enfermera bilingüe con conocimientos en la terminología utilizada en los instrumentos.

A continuación, se llevó a cabo una reunión entre las tres traductoras, en la que se discutieron las discrepancias entre las dos traducciones de cada instrumento y se obtuvo por consenso una primera versión en castellano de cada una de las herramientas.

Tras ello, dos nuevas traductoras bilingües, cuya lengua materna era el castellano y sin conocimiento previo de los cuestionarios originales, realizaron de manera independiente una retrotraducción al inglés de la primera versión en castellano de los instrumentos. La primera traductora era enfermera con conocimiento en el área de cuidado a la familia; la segunda, una enfermera bilingüe con una amplia experiencia en traducción y adaptación de instrumentos. Tras este paso se obtuvieron dos versiones traducidas al inglés para cada uno de los instrumentos.

Finalmente, se conformó un comité compuesto por todas las traductoras involucradas en las etapas anteriores y los autores del proyecto. Este comité comparó las versiones retrotraducidas entre sí y con las versiones originales en inglés de los instrumentos con el fin de evaluar la correspondencia entre ambas en términos de redacción, significado y relevancia. A partir de este análisis se identificaron y discutieron las diferencias semánticas y sintácticas, realizando los ajustes necesarios en la versión en castellano. Como resultado de este proceso, se obtuvieron las versiones prefinales en castellano de los instrumentos. De esta manera, los cuestionarios ICE-FPSQ y HCPP-OFSQ pasaron a denominarse en español: Cuestionario sobre la percepción familiar del apoyo recibido por los profesionales de la salud (CAR-FAM) y Cuestionario sobre la percepción de los profesionales de la salud del apoyo ofrecido a la familia (CAO-FAM), respectivamente.

*Fase 3. Evaluación de la aplicabilidad y comprensión de la versión adaptada*

Se conformó un panel de expertos con 10 enfermeras de las cuales cinco tenían un perfil asistencial y cinco, académico. El objetivo fue evaluar la relevancia y la claridad de ambos instrumentos (CAR-FAM y CAO-FAM) mediante una escala tipo Likert de cuatro puntos: 1 nada relevante/nada claro, 2 poco relevante/poco claro, 3 bastante relevante/bastante claro, y 4 muy relevante/muy claro^24^. En caso de que alguno de los ítems fuera calificado con una puntuación de 1 o 2, se solicitó a los expertos proponer una expresión alternativa.

Posteriormente, se estimó la validez de contenido de ambos cuestionarios. Para la relevancia y claridad de cada ítem, se calculó el *Item-Content Validity Index* (I-CVI), siendo la razón entre el número de expertos que puntuaron 3 o 4 en la escala Likert y el número total de expertos. Para la relevancia y claridad de la escala total, se calculó el *Scale-Content Validity Index Average* (S-CVI/Ave), siendo la razón entre la suma de los I-CVI de todos los ítems del instrumento y el número total de ítems^24^. Además, se calculó el coeficiente Kappa (K*) para evitar la posibilidad de coincidencia por azar entre los expertos.

*Fase 4. Estudio piloto*

Tras la evaluación del panel de expertos, se llevó a cabo un estudio piloto con el objetivo de evaluar la aplicabilidad y la comprensión de los ítems de ambas escalas en las poblaciones diana. El CAO-FAM se aplicó a una muestra de 17 enfermeras pertenecientes la Clínica San Miguel, en Pamplona. Los criterios de inclusión fueron: a) estar trabajando en el momento del estudio, b) tener contacto con los familiares de los pacientes y c) tener fluidez en el idioma español. El CAR-FAM se aplicó a una muestra de 17 familiares de pacientes atendidos en dicho centro hospitalario. Los criterios de inclusión fueron: a) que su familiar estuviera siendo atendido en el centro hospitalario, b) ser mayor de edad y c) tener fluidez en el idioma español.

Tras la aprobación del comité de ética de la investigación del centro hospitalario, las supervisoras de las unidades implicadas facilitaron el acceso a los potenciales participantes profesionales y familiares. Se llevó a cabo un único encuentro con cada participante, durante el cual un miembro del equipo investigador explicó, de forma oral y por escrito el objetivo del estudio y la naturaleza voluntaria de su participación. Posteriormente, se obtuvo el consentimiento informado por escrito de cada participante y, a continuación, se procedió a la recogida de datos, tal y como se detalla a continuación.

En primer lugar, se administró el instrumento a cada participante y el investigador anotó el tiempo estimado para completarlo. En segundo lugar, mediante técnicas de entrevista cognitiva^25^ se evaluó cómo los participantes procesaban e interpretaban las preguntas al completar el cuestionario^26^. Empleando las siguientes preguntas se analizó:

- la dificultad, ¿Le ha resultado difícil completar el cuestionario? Si es así, ¿Por qué?; ¿Le ha generado dudas alguna de las preguntas o términos del cuestionario?;
- la falta o duplicidad de ítems, ¿Cree usted que falta o que sobra alguna pregunta en el cuestionario? ¿Cuál/es?;
- sugerencias, ¿Tiene alguna sugerencia o comentario acerca del instrumento? Por favor, describa. En caso de que los participantes considerasen como confuso, complejo o duplicado alguno de los ítems durante la entrevista cognitiva, se les solicitaron alternativas.

Junto con el instrumento, se administró un cuestionario sociodemográfico.

Las variables sociodemográficas cuantitativas se describieron mediante medias y desviación estándar (DE). En el caso de las enfermeras, estas variables incluyeron edad, antigüedad como enfermera y antigüedad en la unidad actual; en el caso de familiares, se incluyó la edad.

Las variables sociodemográficas cualitativas se expresaron como frecuencias absolutas y porcentajes. Para las enfermeras, dichas variables fueron: sexo, formación académica y profesional, unidad de trabajo actual, tipo de contrato, formación en cuidado centrado en la familia, presencia del enfoque de cuidado centrado en la familia en su unidad, y experiencia personal con un familiar gravemente enfermo. Para los familiares, las variables incluyeron: sexo, nivel educacional, relación con el paciente atendido, si era la primera vez que atendían a su familiar, y servicio en el que se encontraba hospitalizado.

### Consideraciones éticas

Previo al estudio, se obtuvo el permiso de los autores para traducir y utilizar el instrumento original. Asimismo, el estudio fue aprobado por el Comité de Ética de la Investigación (CEI) de la Universidad de Navarra (2023/133) y por la dirección de la Clínica San Miguel donde se llevó a cabo el estudio piloto. Los participantes recibieron información oral y escrita sobre el propósito y diseño del estudio, y todos firmaron un consentimiento informado.

## RESULTADOS

*Fase 1. Adaptación del instrumento para su uso en enfermeras*

Como resultado de esta fase, se obtuvo el instrumento HCPP-OFSQ, cuyo objetivo es evaluar la percepción de las enfermeras respecto al cuidado ofrecido a la familia (Tabla 1).

**Tabla 1:** Versiones originales en inglés de los cuestionarios *Iceland-Family Perceived Support Questionnaire* (ICE-FPSQ) and *Health Care Professionals Perception regarding Offering Family Support Questionnaire* (HCPP-OFSQ)

ICE-FPSQ	HCPP-OFSQ
The nurses on the unit have…	As a nurse, on my unit I have…
1. offered us information and their professional option	1. offered families information and my professional option.
2. provided accessible and easy-to-read literature about the health problem	2. provided accessible and easy-to-read literature about the health problem
3. informed my family about the resources available in the community that have proven to be helpful for families in similar situations	3. informed families about the resources available in the community that have proven to be helpful for families in similar situations
4. provided ideas, information and thoughts in a manner that enabled us to learn from them and reflect on them	4. provided ideas, information and thoughts in a manner that enabled families to learn from them and reflect on them
5. emphasized the use of family rituals to promote family members’ health	5. emphasized the use of family rituals to promote family members’ health
6. offered us family meetings	6. offered family meetings
7. helped family members recognize that our emotional response is valid and helped us to validate and/or normalize family members’ emotional response	7. helped family members recognize that their emotional response is valid and helped them to validate and/or normalize family members’ emotional response
8. encouraged my family to become involved with the health care team in the care of our family member and have offered us caregiver support	8. encouraged families to become involved with the health care team in the care of their family member and have offered them caregiver support
9. encouraged family members to share their illness narratives - not only stories of illnesses and suffering but also stories of strength and resilience	9. encouraged family members to share their illness narratives - not only stories of illnesses and suffering but also stories of strength and resilience
10. drawn out our family strengths	10. drawn out family strengths
11. helped family members understand how our emotional response is related to the family member’s illness	11. helped family members understand how their emotional response is related to the family member’s illness
12. encouraged my family to take a respite from caregiving	12. encouraged families to take a respite from caregiving
13. been aware of the impact family members can have on one another, on the patient’s well-being, and on the illness itself	13. been aware of the impact family members can have on one another, on the patient’s well-being, and on the illness itself
14. looked for the family’s strengths and opportunities to commend family members when their strengths have been revealed	14. looked for the family’s strengths and opportunities to commend family members when their strengths have been revealed

*Fase 2. Traducción y adaptación transcultural del instrumento*

*Traducción*. Tras la traducción se obtuvo la versión española de los instrumentos CAR-FAM (del ICE-FPSQ) y CAO-FAM (del HCPP-OFSQ). Aunque en ambas versiones la mayoría de los ítems eran equivalentes a los originales, los traductores realizaron algunas modificaciones sintácticas y semánticas para mejorar la comprensión y adaptación cultural (Tabla 2). Los objetivos de esas modificaciones fueron la adaptación del inglés al español (ítem 2), el uso de expresiones más comunes en el contexto español (ítems 5 y 10), y la mejora de la comprensión del término *reuniones familiares* (ítems 6 y 14). En relación con las modificaciones sintácticas, en el cuestionario CAR-FAM únicamente se realizaron cambios en los ítems 3 y 7, en los que se empleó la primera persona del plural, elaborando así una expresión más natural en el contexto español.

**Tabla 2:** Proceso de traducción

Proceso de traducción CAR-FAM		
*Modificaciones sintácticas*		
ICE-FPSQ	CAR-FAM	Síntesis I
3. *informed my family about the resources available in the community that have proven to be helpful for families in similar situations*	T1. han informado a mi familia sobre los recursos disponibles en la comunidad que han demostrado ser útiles para familias en situaciones similares	Nos han informado sobre los recursos disponibles en la comunidad que han demostrado ser útiles para familias en situaciones similares
	T2. informado sobre los recursos disponibles en la comunidad que son de ayuda para familias en situaciones similares	
7. *helped family members recognize that our emotional response is valid and helped us to validate and/or normalize family members’ emotional response*	T1. nos han ayudado a reconocer que nuestra respuesta emocional es válida y nos han ayudado a validar y/o normalizar la respuesta emocional de nuestros familiares	Nos han ayudado, a los miembros de la familia, a reconocer que nuestras emociones son válidas, y nos han ayudado a validar y/o normalizar la respuesta emocional de nuestros familiares
	T2. ayudado, a los miembros de la familia, a reconocer que nuestras emociones son válidas, y nos han ayudado a validar y/o normalizar la respuesta emocional de los miembros de la familia	
*Modificaciones semánticas*		
ICE-FPSQ	CAR-FAM	Síntesis I
2. *provided accessible and easy-to-read literature about the health problem*	T1. han proporcionado literatura accesible y de fácil lectura sobre el problema de salud.	Nos han proporcionado información accesible y fácil de leer sobre el problema de salud.
	T2. proporcionado información accesible y fácil de leer sobre el problema de salud	
5. *emphasized the use of family rituals to promote family members’ health*	T1. han enfatizado el uso de rituales familiares para promover la salud de los miembros de la familia.	Nos han recomendado mantener nuestras costumbres familiares para promover la salud de los miembros de la familia.
	T2. Han enfatizado el uso de rutinas familiares para mejorar la salud de los miembros de la familia	
6. *offered us family meetings*	T1. han ofrecido reuniones familiares.	Nos han ofrecido reuniones familiares (reuniones entre los profesionales de la salud y el paciente y/o familia)
	T2. Han ofrecido entrevistas familiares.	
10. *drawn out our family strengths*	T1. han destacado las fortalezas de nuestra familia.	Han destacado las fortalezas de nuestra familia.
	T2. subrayado nuestras fortalezas familiares	
14. *looked for the family’s strengths and opportunities to commend family members when their strengths have been revealed*	T1. han buscado las fortalezas de la familia y las oportunidades para elogiar a los miembros de la familia cuando sus fortalezas han sido reveladas.	Han identificado nuestros puntos fuertes como familia, y han aprovechado las oportunidades para elogiar nuestras fortalezas
	T2. identificado nuestros puntos fuertes como familia, y han aprovechado las oportunidades para elogiar nuestras fortalezas	
*Proceso de traducción CAO-FAM*		
*Modificaciones semánticas*		
ICE-SPOSQ	CAO-FAM	Síntesis I
2. *I provide accessible and easy-to-read literature about the health problem to the family*	T1. he proporcionado literatura accesible y de fácil lectura sobre el problema de salud	he proporcionado información accesible y fácil de leer sobre el problema de salud
	T2. proporcionado información accesible y fácil de leer sobre el problema de salud	
5. *I emphasize the use of family rituals to promote family members’ health*	T1. he enfatizado el uso de rituales familiares para promover la salud de los miembros de la familia.	he recomendado a las familias mantener sus costumbres para promover la salud de los miembros de la familia.
	T2. he enfatizado el uso de rutinas familiares para mejorar la salud de los miembros de la familia	
6. *I offer family meetings*	T1. he ofrecido reuniones familiares.	he ofrecido reuniones familiares (reuniones entre los profesionales de la salud y el paciente y/o familia).
	T2. he ofrecido entrevistas familiares	
10. *I drawn out family strengths*	T1. he resaltado las fortalezas de la familia.	he resaltado las fortalezas de la familia
	T2. subrayado las fortalezas familiares	
14. *I looked for the family’s strengths and opportunities to commend family members when their strengths have been revealed*	T1. he buscado las fortalezas de la familia y las oportunidades para elogiar a los miembros de la familia cuando sus fortalezas han sido reveladas.	he identificado sus puntos fuertes como familia, y he aprovechado las oportunidades para elogiar sus fortalezas.
	T2. identificado los puntos fuertes de las familias, y he aprovechado las oportunidades para elogiar sus fortalezas cuando se han manifestado	

*Retrotraducción.* Las versiones obtenidas en el proceso de retrotraducción fueron muy similares a los cuestionarios originales. El equipo de traductores realizó modificaciones sintácticas en los ítems 11 y 14 en ambos instrumentos con el fin de facilitar la lectura, sin alterar el sentido original de los ítems (Tabla 3).

**Tabla 3:** Proceso de retrotraducción

Proceso de retrotraducción (CAR-FAM)		
*Modificaciones sintácticas*		
ICE-FPSQ	CAR-FAM	Síntesis II
11. *helped family members understand how our emotional response is related to the family member’s illness*	T1. *helped us understand how our emotional response influences our family member’s illness.*	Nos han ayudado a entender cómo influyen nuestras emociones en la enfermedad.
	T2. *They have helped us to understand how our emotional response influences the disease of our relative*	
14. *looked for the family’s strengths and opportunities to commend family members when their strengths have been revealed*	T1. *identified our strengths as a family, and took opportunities to commend our strengths.*	Han identificado nuestros puntos fuertes como familia, y han elogiado nuestras fortalezas
	T2. *They have identified our strengths as family and have taken the opportunity to praise them*	
*Proceso de retrotraducción (CAO-FAM)*		
*Modificaciones sintácticas*		
ICE-SPOSQ	CAO-FAM	Síntesis II
11. I *help family members understand how their emotional response is related to the family member’s illness*	T.1. helped family members to *understand how their emotional response influences their relative´s illness.*	He ayudado a los miembros de la familia a entender cómo influyen sus emociones en la enfermedad de su familiar
	T.2. *I have helped family members to understand how their emotional response influences the disease of their relative*	
14. *I looked for the family’s strengths and opportunities to commend family members when their strengths have been revealed*	T1. *identify the strengths each family has and take opportunities to commend their strengths.*	He identificado sus puntos fuertes como familia, y he elogiado sus fortalezas
	T2. *I have identified their strengths as family and I have taken the opportunity to praise them*	

*Fase 3. Evaluación de la aplicabilidad y comprensión de la versión adaptada*

Ambos cuestionarios superaron los límites aceptables de relevancia (K*>0,6, I-CVI>0,78, S-CVI/Ave>0,9)^22^. Todos los ítems alcanzaron valores de I-CVI superiores a 0,78, mientras que el S-CVI/Ave obtuvo un valor de 0,958 en ambos instrumentos. Además, los valores de K* fueron superiores a 0,6.

En cuanto a la claridad, varios ítems (1, 2, 5, 7, 12 y 13 en el CAR-FAM, y 1, 5, 12 y 13 en el CAO-FAM) presentaron valores inferiores a 0,60 en I-CVI y K*, por lo que se realizaron modificaciones para mejorar su claridad.

- En el CAR-FAM:
  - El ítem 5: “Nos han recomendado el uso de costumbres familiares” fue sustituido por “Nos han recomendado mantener nuestras costumbres familiares”, siendo una expresión más clara y natural en el contexto español.
- En el CAO-FAM:
  - El ítem 2: “he proporcionado información accesible y fácil de leer” fue sustituido por “he proporcionado material informativo accesible y fácil de leer” facilitando la comprensión de la expresión.
  - Para el ítem 7: se simplificó la oración “he ayudado a los miembros de la familia a reconocer que sus emociones son válidas y a normalizar la respuesta emocional de sus familiares” por “he ayudado a los miembros de la familia a validar y normalizar sus emociones y la de sus familiares” procurando una redacción del ítem más directa y concisa.
  - El ítem 13: “he sido consciente del impacto que los familiares pueden tener entre sí, en el bienestar del paciente y en la propia enfermedad” fue sustituido por “he sido consciente de cómo pueden influir los miembros de la familia entre sí, en la enfermedad, y en el bienestar del paciente”. Añadiendo el término “influir” y cambiando el orden de redacción del ítem se ha conseguido una secuencia más clara y lógica.

Siguiendo las indicaciones de Polit y col^24^, se llevó a cabo un nuevo papel de expertos, compuesto por tres miembros que ya participaron en el primer panel, para evaluar nuevamente la claridad del instrumento.

Tras los cambios, todos los ítems obtuvieron valores I-CVI y K* de 1 en cuanto a claridad, excepto los ítems 1, 2 y 13 (con valores I-CVI = 0,333; y k* <0,4), resultando en un S-CVI/Ave de 0,77 para el CAR-FAM y de 0,88 para el CAO-FAM (Tabla 4).

**Tabla 4:** Propiedades psicométricas (claridad y relevancia) del CAR-FAM y del CAO-FARM tras el segundo panel de expertos

CAR-FAM	Relevancia		Claridad	
	I-CVI	K*	I-CVI	K*
Por favor, marque una “x” en la casilla que mejor describa su percepción sobre el apoyo familiar recibido por parte de los profesionales de la salud. Por favor, tenga en cuenta que no hay respuestas correctas o incorrectas	1	1	1	1
Casi Nunca/ Raramente/ Ocasionalmente/ Frecuentemente/ Casi Siempre	1	1	1	1
Las enfermeras en la unidad…	1	1	1	1
Nos han ofrecido información y opinión profesional	1	1	0,333	<0,4
Nos han proporcionado material informativo accesible y fácil de leer sobre el problema de salud	1	1	0,333	<0,4
Nos han informado sobre los recursos disponibles en la comunidad que han sido útiles para familias en situaciones similares	1	1	1	1
Nos han aportado ideas, información y otros puntos de vista que nos han permitido aprender y reflexionar sobre ellos	1	1	1	1
Nos han recomendado mantener nuestras costumbres familiares para promover la salud de los miembros de la familia	1	1	0,3333	<0,4
Nos han ofrecido reuniones familiares (reuniones entre los profesionales de la salud y el paciente y/o familia)	1	1	1	1
Nos han ayudado a validar y/o normalizar nuestras emociones y/o las de nuestros familiares	1	1	1	1
Nos han animado a involucrarnos con el equipo sanitario en el cuidado de nuestro familiar, y nos han ofrecido apoyo como cuidadores	1	1	1	1
Nos han animado a compartir nuestra experiencia, no solo a hablar de la enfermedad y el sufrimiento, sino también de nuestras fortalezas y resiliencia	1	1	1	1
Han destacado las fortalezas de nuestra familia	1	1	1	1
Nos han ayudado a entender cómo influyen nuestras emociones en la enfermedad	1	1	1	1
Nos han animado a tomarnos un respiro en el cuidado	1	1	1	1
Han sido conscientes de cómo pueden influir los miembros de la familia entre sí, en la enfermedad, y en el bienestar del paciente	1	1	0,333	<0,4
Han identificado nuestros puntos fuertes como familia, y han elogiado nuestras fortalezas	1	1	1	1
*S-CVI/Ave*	*1*		*0,770588*	
*CAO-FAM*				
Por favor, marque con una “x” en la casilla que mejor describa su percepción sobre el apoyo profesional ofrecido a la familia. Por favor, tenga en cuenta que no hay respuestas correctas o incorrectas	1	1	1	1
Casi Nunca/ Raramente/ Ocasionalmente/ Frecuentemente/ Casi Siempre	1	1	1	1
Como enfermera, en mi unidad…	1	1	1	1
He ofrecido a las familias información y opinión profesional	1	1	0,333	<0,4
He proporcionado material informativo accesible y fácil de leer sobre el problema de salud	1	1	0,333	<0,4
He informado a las familias sobre los recursos disponibles en la comunidad que han sido útiles para familias en situaciones similares	1	1	1	1
He proporcionado ideas, información y otros puntos de vista que han permitido a las familias aprender y reflexionar sobre ellos	1	1	1	1
He recomendado a las familias mantener sus costumbres para promover la salud de los miembros de la familia	1	1	1	1
He ofrecido reuniones familiares (reuniones entre los profesionales de la salud y el paciente y/o familia)	1	1	1	1
He ayudado a los miembros de la familia a validad y normalizar sus emociones y la de sus familiares	1	1	1	1
He animado a las familias a involucrarse con el equipo sanitario en el cuidado de su familiar, y les he ofrecido apoyo como cuidadores	1	1	1	1
He animado a los familiares a compartir su experiencia, no solo a hablar de la enfermedad y el sufrimiento, sino también de sus fortalezas y resiliencia	1	1	1	1
He destacado las fortalezas de las familias	1	1	1	1
He ayudado a los miembros de la familia a entender cómo influyen sus emociones en la enfermedad de su familiar	1	1	1	1
He animado a las familias a tomarse un respiro en el cuidado	1	1	1	1
He sido consciente de cómo pueden influir los miembros de la familia entre sí, en la enfermedad, y en el bienestar del paciente	1	1	0,333	<0,4
He identificado sus puntos fuertes como familia, y he elogiado sus fortalezas	1	1	1	1
*S-CVI/Ave*	*1*		*0,88235*	

I-CVI: *Item content validity index* (Índice de validez de contenido), S-CVI/Ave: *Scale content validity index average* (Índice de validez de contenido de la escala/promedio). K*: índice de Kappa modificado.

*Fase 4. Estudio piloto*

El estudio piloto del CAR-FAM se aplicó a una muestra de 17 familiares de pacientes (9 mujeres y 8 hombres), con edad media de 49 años. Diez tenían a su familiar ingresado en urgencias y siete en una planta de hospitalización. Seis de los participantes eran cónyuges del paciente, cinco padres/madres, cuatro hermanas/hermanos y dos hijos/hijas (Tabla 5).

Por otro lado, el cuestionario CAO-FAM fue aplicado a una muestra de 17 enfermeras, la mayoría mujeres (94%), con una media de edad de 39,82 años y una experiencia laboral promedio de 16,41 años. Diez trabajaban en planta de hospitalización, seis en urgencias y una en quirófano. Doce enfermeras consideraban que en su unidad se llevaba a cabo un enfoque de cuidado orientado a la familia, y cuatro habían recibido algún tipo de formación en el modelo de cuidado centrado en la familia (Tabla 5).

**Tabla 5:** Características sociodemográficas de los participantes en el estudio piloto

Características	n (%)
*Familiares*	
Edad (años)*	49,2 (17,45); 18-72
*Sexo*	
Mujer	9 (52,9)
Hombre	8 (47,05)
*Nivel educacional*	
Educación básica	5 (29,4)
Formación profesional	3 (17,6)
Grado universitario	7 (41,1)
Postgrado universitario	2 (11,7)
*¿Cuál es su relación con su familiar atendido? Usted es…*	
Padre/madre	5 (29,4)
Cónyuge	6 (35,2)
Hermana/Hermano	4 (23,5)
Hija/Hijo	2 (11,7)
*¿Es la primera vez que ingresan/atienden a su familiar?*	
Sí, nunca antes había sido ingresado/atendido	5 (29,4)
No, hace más de un año también fue ingresado/atendido	7 (41,1)
No, en el último año también ha sido ingresado/atendido	5 (29,4)
*¿En qué servicio se encuentra ingresado/atendido su familiar?*	
Planta de hospitalización	7 (41,1)
Urgencias	10 (58,8)
*Enfermeras*	
Edad (años)*	39,82 (13,46); 23-60
*Sexo*	
Mujer	16 (94,1)
Hombre	1 (5,9)
*Formación académica*	
Graduada/Diplomada	13 (76,4)
Máster	4 (23,5)
*Formación Profesional*	
Especialidad	2 (11,7)
Experto	3 (17,6)
Otro	1 (5,9)
Ninguno	11 (64,7)
Antigüedad como enfermera (años)*	16,41 (12,17); 1-35
*Unidad de trabajo actual*	
Planta de hospitalización	10 (58,8)
Urgencias	6 (435,2)
Quirófano	1 (5,9)
*Enfermeras*	
Antigüedad en la unidad actual (años)*	11,88 (10,11); 1-34
*Tipo de contrato*	
Eventual/Interino	(23,5)
Fijo laboral	13 (76,4)
¿Ha recibido algún tipo de formación en Cuidado Centrado en la Familia? (sí)	(23,5)
¿En su lugar de trabajo hay un enfoque de Cuidado Centrado en la Familia? (sí)	12 (70,5)
¿Ha estado algún miembro de su familia gravemente enfermo y ha necesitado cuidado profesional? (sí)	16 (94,1)

*Media (DE); rango.

El tiempo promedio de respuesta para ambos cuestionarios fue de dos minutos. Sin embargo, un familiar y una enfermera requirieron un tiempo ligeramente mayor, ambos completando el cuestionario en cuatro minutos.

Los familiares no presentaron dificultades para completar el CAR-FAM; seis de ellos destacaron la facilidad de comprensión del instrumento, y ninguno reportó dificultades. Respecto al CAO-FAM, cinco enfermeras señalaron que la comprensión del cuestionario era sencilla, y dos de ellas resaltaron la rapidez de cumplimentación.

## DISCUSIÓN

Tras el proceso de traducción y adaptación transcultural del ICE-FPSQ, se ha obtenido la primera versión española del instrumento, CAR-FAM, equivalente a la versión original a nivel semántico y conceptual. Este paso es fundamental para la evaluación futura de las propiedades psicométricas del instrumento, permitiendo contar con una versión válida y fiable adaptada al contexto español. Además, se ha desarrollado una nueva versión del instrumento, el CAO-FAM, que posibilita medir la percepción de las enfermeras sobre el apoyo ofrecido a la familia, permitiendo evaluar un mismo constructo desde dos perspectivas complementarias: la de las enfermeras y la de los familiares.

Conocer y analizar la percepción de las enfermeras y los familiares sobre el apoyo y cuidado profesional facilita la identificación de posibles convergencias y divergencias, información valiosa para detectar áreas de mejora organizativa y asistencial, así como necesidades no cubiertas o discrepancias en las expectativas de apoyo. Esta aproximación también permite profundizar en los elementos clave para fomentar una colaboración efectiva entre profesionales y familias, basada en la confianza mutua y la comprensión de sus respectivas perspectivas^27^. Consecuentemente, puede contribuir a la implementación de intervenciones de apoyo dirigidas tanto al paciente como a su familia, promoviendo una cultura de colaboración que mejora los resultados para los pacientes, sus familiares y los propios profesionales^19^.

La inclusión de ambas perspectivas, la de las enfermeras y la de las familias, fortalece la validez de contenido de los instrumentos^28^, ya que permite evaluar de manera más completa el fenómeno del apoyo familiar. Como señala la revisión sistemática de Jemes Campaña y col^28^, la mayoría de los estudios se han centrado únicamente en la percepción de las familias, aunque cada vez son más los que incluyen también la visión de los profesionales. Considerar la perspectiva de ambos grupos proporciona evaluaciones más completas y mejora la calidad metodológica de los estudios, al reflejar de manera más precisa la realidad del cuidado y apoyo ofrecido y permitiendo realizar una comparación entre poblaciones^28^^,^^29^.

El proceso de traducción y adaptación del ICE-FPSQ al contexto español siguió la guía de Sousa y Rojjanasrirat^22^, reconocida por su metodología sistemática y rigurosa. Esta metodología propone la participación de dos traductores independientes en cada fase y la intervención de un tercero para sintetizar las versiones, lo que incrementa la fiabilidad de la traducción y asegura su equivalencia lingüística y cultural. Epstein y col^30^ llevaron a cabo una revisión de diversas guías de traducción y adaptación de instrumentos y, aunque no hallaron un consenso general, identificaron como pasos recurrentes la conformación de comités de expertos y la retrotraducción, ambos contemplados en la guía de Sousa y Rojjanasrirat^22^. Además, subrayaron la importancia de llevar a cabo un proceso riguroso y sistemático para la adaptación transcultural y la implicación de traductores bilingües especializados en el proceso^30^.

La metodología sistemática seguida permitió abordar y resolver las principales dificultades en la traducción y adaptación de los instrumentos, garantizando la equivalencia conceptual y lingüística mediante el consenso entre los tres traductores. En particular, los ítems 5 y 6 presentaron los mayores desafíos debido a la complejidad de los términos “*family rituals*” y “*family meetings*”. Tras el análisis de las divergencias, se alcanzó un acuerdo para traducirlos como “costumbres familiares” y “reuniones familiares”, respectivamente; en este último caso, se incorporó además una definición complementaria con el fin de evitar ambigüedades. Esta dificultad también fue descrita por Freudiger y col^18^, que llevaron a cabo la traducción y validación del ICE-FPSQ al contexto alemán. En dicho estudio, el ítem 6 (*the nurses on the unit have offered us family meetings*) generó confusión, ya que su interpretación se inclinaba más a “reuniones interprofesionales” entre un miembro del equipo y un familiar, en lugar de a encuentros grupales dirigidos a toda la familia. Para mitigar este problema, los autores proponían el uso de términos más concretos de manera que se evite la libre interpretación del término. Por ello, siguiendo esta misma estrategia, la inclusión de una definición detallada de “reuniones familiares” en la versión en español contribuye a reducir la posibilidad de interpretaciones erróneas y garantiza una mejor comprensión del ítem en el contexto clínico.

Un aspecto fundamental en la adaptación de instrumentos es la evaluación de la relevancia y claridad de los ítems, que en este estudio se ha abordado mediante la implementación de un panel de expertos. Este panel, compuesto por profesionales de la salud con experiencia en metodología de investigación y traducción de instrumentos, desempeñó un papel crucial en la revisión de los ítems del CAR-FAM y CAO-FAM. Su retroalimentación no solo garantizó que los ítems fueran culturalmente pertinentes, sino que también permitió ajustar el lenguaje de los ítems para que reflejaran mejor las experiencias vividas por los familiares y profesionales en el contexto español. Así, la inclusión de esta evaluación de expertos fortalece la validez de contenido de los instrumentos, asegurando que sean herramientas precisas y adecuadas para medir la percepción del cuidado desde ambas perspectivas, la perspectiva de los familiares y de los profesionales^22^.

Como limitaciones de este trabajo cabe mencionar que la realización del estudio piloto, así como las entrevistas cognitivas, han sido llevadas a cabo íntegramente en un único centro, con una muestra suficiente pero no elevada, lo que podría afectar a la validez externa de los instrumentos. No obstante, considerando la favorable comprensibilidad demostrada por los participantes del estudio piloto, no se consideró necesaria la ampliación de la muestra o la inclusión de otros centros. Durante el proceso de validación de contenido de la versión dirigida a profesionales (CAO-FAM) se priorizó la coherencia conceptual y la equivalencia métrica entre CAO-FAM y CAR-FAM; es importante señalar que, aunque facilita comparaciones, ha podido limitar la capacidad para capturar la complejidad de la evaluación del apoyo familiar desde la perspectiva profesional. Por otro lado, el proceso de traducción y retrotraducción de los instrumentos se ha realizado en el marco del español estándar o neutro. Esta elección metodológica podría tener implicaciones en la validez de los instrumentos cuando se apliquen en contextos lingüísticos caracterizados por variantes dialectales significativas o variedades del español marcadamente diferenciadas.

En conclusión, se han desarrollado los instrumentos CAR-FAM y CAO-FAM, versiones traducidas y adaptadas al contexto español de los instrumentos originales ICE-FPSQ y HCCP-OFSQ, respectivamente. Estas herramientas permitirán evaluar la percepción del apoyo familiar desde la perspectiva tanto de los familiares como de los profesionales de la salud.

El proceso sistemático de traducción y retrotraducción, junto con los resultados obtenidos con índices de claridad y relevancia superiores al límite aceptable, respaldan la robustez de la validez de contenido de los instrumentos y su idoneidad para llevar a cabo un estudio de validación con una muestra más amplia. Este próximo paso será fundamental para medir la consistencia interna y determinar las restantes propiedades psicométricas, entre ellas, la validez interna, validez estructural y análisis factorial de las versiones españolas, garantizando así su aplicabilidad en el contexto clínico y de investigación.

## Data Availability

Se encuentran disponibles bajo petición a la autora de correspondencia.

## ANEXO I

**Table:** Descripción de los estudios del ICE-FPSQ y calidad de las propiedades psicométricas reportadas

Autores	Idioma	Validez de contenido	Validez estructural	Prueba de hipótesis	Validez Transcultural	Consistencia interna	Fiabilidad
Año	Muestra						
País	Contexto						
Sveinbjarnardottir et al.	Inglés	+	+	+	+	+	+
(2012)	335 F						
Suecia	Oncología pediátrica						
Bruce et al.	Sueco	+	+		+	+	+
(2016)	97 F						
Suecia	Cardiopatía congénita infantil						
Eggenberger y Sanders	Inglés					+	
(2016)	35 F						
Estados Unidos	UCI						
Dieperink et al.	Danés e inglés				+	+	+
(2017)	93 F						
Dinamarca y Australia	139 P						
Konradsen et al.	Danés		?		+	+	+
(2018)	110 F/P						
Dinamarca							
Lemos et al.	Portugués	+	+		+	+	+
(2022)	237 F						
Portugal	Pediatría						
Freudiger et al.	Alemán	+	+		+	+	
(2024)	77 F						
Suiza	UCI						

F: familia; P: pacientes. La calidad de los resultados se calificó como positiva (+), negativa (-) o indeterminada (?) según los criterios de Mokkink y col*. El error de medición y las propiedades de respuesta se omiten en la tabla porque ningún estudio las evaluó; además, ningún resultado fue evaluado con calidad negativa.

* Mokkink LB, Elsman EBM, Terwee CB. COSMIN guideline for systematic reviews of patient-reported outcome measures version 2.0. Qual Life Res 2024; 33(11): 2929-2939. https://doi.org/10.1007/s11136-024-03761-6
